# EFFECTS OF SMARTHEALTH INTERVENTION ON DEMENTIA CAREGIVERS’ REACTIONS AND EMOTIONS: A PILOT STUDY

**DOI:** 10.1093/geroni/igae098.4171

**Published:** 2024-12-31

**Authors:** Eunjung Ko, Kathy Wright, Kristina Gordon, John Stankovic, Hongning Wang, Ashley Gao, Peng Wang, Karen Rose

**Affiliations:** New York University, New York, New York, United States; The Ohio State University, Columbus, Ohio, United States; The University of Tennessee Knoxville, Knoxville, Tennessee, United States; University of Virginia, Charlottesville, Virginia, United States; University of Virginia, University of Virginia, Virginia, United States; William & Mary, Williamsburg, Virginia, United States; University of Virginia, Charlottesville, Virginia, United States; The Ohio State University, Columbus, Ohio, United States

## Abstract

Dementia care can cause emotional distress among caregivers. As technology advances, innovative interventions to alleviate stress in this group have emerged, highlighting the need for more investigation. We aimed to develop a deep learning-based smarthealth intervention using acoustic monitoring and ecological momentary assessment to detect stress and deliver stress management messages. We also intended to identify the intervention effects on the reactions and emotions of caregivers of people living with dementia. During the four-month intervention period, we collected data about participants’ emotional status and reactions to their care recipients’ symptoms through pre- and post-questionnaires, periodic phone surveys, and semi-structured interviews. Descriptive analysis, Wilcoxon signed-rank test, and abductive thematic analysis were used. Ten of 11 participants completed the study. The average age was 60 years, and participants reported providing care for approximately three years. Participants were predominently female (72.3%), White (81.8%), and spouses (63.6%). Pre-and post-questionnaires showed significant changes in caregivers’ perceived frequency of care recipients’ disruptive symptoms (p=.026) and their reactions to these symptoms (p=.006), but not in other emotional-related variables. Although answers to periodic phone surveys varied, participants tended to consider self-care when they reported better physical health and less stress and depressive mood. Interviews revealed seven themes—affective attitudes, burden, ethicality, intervention adherence, intervention coherence, perceived effectiveness, and suggestions—containing 27 categories. While participants’ feedback on our smarthealth intervention was mixed, the intervention improved participants’ awareness of emotions and caregiving situations. The findings suggest the potential benefits of the smarthealth intervention on caregivers’ awareness of emotions and self-care.

